# Influenza virus-like particle vaccine containing both apical membrane antigen 1 and microneme-associated antigen proteins of *Plasmodium berghei* confers protection in mice

**DOI:** 10.1186/s12865-022-00494-4

**Published:** 2022-04-25

**Authors:** Min-Ju Kim, Ki-Back Chu, Hae-Ji Kang, Keon-Woong Yoon, Dong-Hun Lee, Su-Hwa Lee, Eun-Kyung Moon, Fu-Shi Quan

**Affiliations:** 1 Department of Biomedical Science, Graduate School, Kyung Hee University, Seoul, 02447 Republic of Korea; 2 Department of Medical Zoology, Kyung Hee University School of Medicine, Seoul, 02447 Republic of Korea; 3 Department of Medical Research Center for Bioreaction to Reactive Oxygen Species and Biomedical Science Institute, School of Medicine, Graduate School, Kyung Hee University, 26, Kyungheedae-ro, Dongdaemun-gu, Seoul, 02447 Republic of Korea

**Keywords:** *Plasmodium berghei*, Apical membrane antigen 1, Microneme, Virus-like particles, Vaccine

## Abstract

**Background:**

Apical membrane antigen 1 (AMA1) and microneme-associated antigen (MIC) of *Plasmodium* parasites are important factors involved in host cell invasion.

**Methods:**

In this study, influenza VLP vaccines containing both codon-optimized AMA1 and MIC were generated and the vaccine efficacy was evaluated in mice.

**Results:**

VLPs vaccine immunization elicited higher levels of parasite-specific IgG and IgG2a antibody responses in sera. CD4^+^ and CD8^+^ T cells and germinal center B cells in blood, inguinal lymph nodes (ILN) and spleen were found to be significantly increased. Importantly, VLPs vaccination significantly reduced the levels of pro-inflammatory cytokines IFN-γ and TNF-α, decreased parasitemia in blood, resulting in lower body weight loss and longer survival time compared to control.

**Conclusion:**

These results indicated that VLPs containing *P. berghei* AMA1 and MIC could be a candidate for malaria blood-stage vaccine design.

**Supplementary Information:**

The online version contains supplementary material available at 10.1186/s12865-022-00494-4.

## Background

Global malaria incidence rates have been declining over the past few decades, but malaria continues to remain one of the most devastating infectious diseases affecting the globe today. In the year 2018, malaria was responsible for approximately 405,000 deaths with a vast majority of these occurring in various parts of Africa [1, 2]. Developing an efficacious malaria vaccine is a monumental task and to date, a licensed malaria vaccine remains unavailable simply due to the complex nature of the *Plasmodium* parasite’s life cycle. Notably, *Plasmodium* parasites undergo several developmental stages and express over 5000 genes encoding multitudes of proteins that could be utilized as vaccine candidates [3]. In 2021, the World Health Organization (WHO) recommended the use of RTS,S/AS01 recombinant protein vaccine to immunize children residing in malaria-endemic parts of the world [4]. However, given the 26–50% protection rate demonstrated by this vaccine in children during the phase 3 clinical trial [5, 6], there is a pressing need to improve vaccine design strategy and develop a highly efficacious malaria vaccine.

A novel strategy targeting multiple antigens with an alternative vaccine is urgently needed.

Vaccines targeting the antigens of merozoites can inhibit the replication of *Plasmodium* parasites, which ultimately contributes to limiting the number of circulating blood-stage parasites and their transmission [3]. Apical membrane antigen 1 (AMA1) is one of the several proteins expressed on the surface of merozoites and plays a crucial role in the parasitic invasion of erythrocytes [7]. Recently, we generated influenza virus-like particles expressing codon-optimized AMA1 of *Plasmodium berghei* and found that VLPs vaccines containing AMA1 showed parasite-specific IgG antibody responses and T cell responses, contributing to diminished parasitemia in the blood of mice [8]. Glycosylphosphatidylinositol-anchored micronemal antigens from *P. falciparum* and *P. vivax* have been characterized and their potential as malaria vaccine candidates have been investigated [9, 10]. Another study characterized a novel *P. falciparum* microneme-associated antigen that functions as an adhesin required for erythrocyte infection [11]. Similar to *Plasmodium* spp., *T. gondii* also expresses micronemal proteins on its surface which contribute to parasite invasion. Attenuated *T. gondii* strain prepared by genetic ablation of the sporozoite protein with an altered thrombospondin repeat, a micronemal protein conserved across the phylum Apicomplexa, resulted in nearly halved parasitic invasion compared to wild-type strain when inoculated into mice [12]. Given their role in the parasite’s infection, constructing a vaccine based on these micronemal antigens could confer significant protection against infection with *Plasmodium* spp. Yet, incorporating these microneme-associated antigens as VLP vaccine components have not been reported to date. Incorporating several antigens into vaccines can improve their protective efficacy. Results of a clinical trial involving hepatitis B vaccine demonstrated that immunizing healthy individuals with single antigen vaccine conferred less protective immunity compared to trivalent vaccine immunization, with dose-sparing effect also being observed from the latter [13]. Based on this rationale, we hypothesized that VLPs simultaneously expressing multiple *Plasmodium* antigens may lead to better protection compared to single antigen AMA1 VLP vaccine.

In this study, influenza virus-like particles containing both AMA1 and microneme-associated antigen (MIC) from *P. berghei* were generated and the vaccine efficacy was evaluated in mice. We found that VLPs vaccine expressing both AMA1 and MIC elicited higher levels of humoral and cellular immune responses, in addition to significantly reducing parasite replication in the blood.

## Materials and methods

### Ethics statement

All experimental methods were carried out in accordance with the guidelines and regulations set out by Kyung Hee University IACUC. All experimental protocols were approved by the Kyung Hee University IACUC (permit number: KHUIBC(SE)-19-034). All of the methods and experimental procedures were carried out in compliance with the ARRIVE guidelines. Immunization and blood collection were performed under mild anesthesia, which was induced and maintained with ketamine hydrochloride and xylazine. All efforts were made to minimize the number of animals used in the experiment as well as their suffering.

### Animals, parasite, cells, and antibodies

Seven-week-old female BALB/c mice were purchased from NARA Biotech (Seoul, Korea). *Plasmodium berghei* ANKA strain was maintained in BALB/c mice and used for challenge infection as described previously [8]. *Spodoptera frugiperda* SF9 cells were used for the production of recombinant baculovirus (rBV), and virus-like particles were maintained using serum-free SF900-II medium (Invitrogen, Carlsbad, CA, USA) as described previously [5]. *Plasmodium berghei*-infected sera from mice were collected through retro-orbital plexus puncture. Horseradish peroxidase (HRP)-conjugated goat anti-mouse immunoglobulin G and its subclasses (IgG, IgG1, IgG2a, and IgG2b) were purchased from Southern Biotech (Birmingham, Alabama, USA).

### *Plasmodium berghei* antigen preparation

*P. berghei* ANKA strain antigen was prepared as described previously [14, 15]. Briefly, *P. berghei*-infected red blood cells (RBCs) were collected from whole blood of mice with parasitemia exceeding 20% by low-speed centrifugation (240 × *g* for 10 min) at 4 °C. Pelleted RBCs were lysed with an equal volume of 0.15% saponin in PBS for 10 min at 37 °C, and the released parasites were pelleted and washed 3 times with PBS (1320×*g* for 1 min, 4 °C). Parasites were sonicated twice at 40 Hz, 30 s each on ice, and stored at − 20 °C until use. *P. berghei* antigen was used as coating antigens for enzyme-linked immunosorbent assay (ELISA) and also as a stimulant during fluorescence-activated cell sorting (FACS).

### Generation of VLPs

Codon-optimized *P. berghei* AMA1 (accession number: XM_672965.2, 1671 bp) and MIC (accession number: XM_034568180.1, 933 bp) genes in pFastBac™ were provided from GenScript (Piscataway, New Jersey, USA). The recombinant plasmids were transformed into DH10Bac competent cells and bacmid DNA was extracted using FavorPrep gel purification Kit (Favorgen, Cheshire, UK). Recombinant baculoviruses (rBV) expressing AMA1, MIC, or M1 were produced as described previously [16]. To produce AMA1 VLPs and MIC VLPs, SF9 cells were co-infected with rBVs expressing AMA1 or MIC with M1 as described previously [16]. M1 VLPs were prepared by infecting SF9 cells with rBVs expressing the M1 protein, and were confirmed using mouse anti-M1 antibody by western. The contents for AMA1 and MIC in VLPs were confirmed using sera from parasite-infected mouse by western blot as described previously [8].

### Immunization and challenge

Seven-week-old female BALB/c mice (n = 10 per group) were randomly grouped as follows: naïve, naïve challenge, VLPs containing both AMA1 and MIC (VLPs(AMA1 + MIC)), and VLPs containing MIC alone, (VLPs(MIC)). Mice were intramuscularly immunized with MIC VLPs (100 ug) only for VLPs(MIC) group, or the mixture of AMA1 VLPs (50 ug) and MIC VLPs (50 ug) for VLPs(AMA1 + MIC) group at weeks 0, 4, and 8. Four weeks after the 2nd boost immunization, mice were challenge-infected with 0.5% of *P. berghei* in 100 μL PBS ($0.5\times {10}^{4}$) by intraperitoneal (IP) injection, as described previously [8]. Five mice from each group were sacrificed at day 6 post-challenge infection for blood, inguinal lymph node (ILN), and spleen sample collection. The remaining mice were observed daily to monitor changes in bodyweight and survival rate. Mice that lost 20% of their body weight were humanely euthanized.

### Antibody responses in sera

Mice sera were collected 4 weeks after prime, 1st boost immunization, and 2nd boost immunization via retro-orbital plexus puncture. Sera from naïve mice were used as a negative control. Antibody responses against *P. berghei* antigen, AMA1 VLPs, and MIC VLPs were determined by ELISA as described [16]. Briefly, 96-well immunoplates were coated with 100 μL of *P. berghei* antigen, AMA1 VLPs, or MIC VLPs in 0.05 M, pH 9.6 carbonate bicarbonate buffer per well at 4 °C overnight at a final concentration of 2, 0.5, and 0.5 μg/ml, each respectively. Afterward, 100 μl of serially diluted serum samples were added into respective wells and incubated at 37 °C for 1.5 h. HRP-conjugated goat anti-mouse IgG, IgG1a, IgG2a, and IgG2b (100 μl/well, diluted 1:2000 in PBS) were used to determine *P. berghei*-specific antibody responses.

### Immune cell responses by flow cytometry

To determine immune cell responses, the levels of T and B cell populations from blood, spleen, and ILN of mice were investigated by flow cytometry as described previously [8]. CD4^+^ and CD8^+^ T cells from the blood were detected after 2nd boost immunization and on day 6 after challenge infection. The levels of CD4^+^, CD8^+^ T cells, and germinal center B cells from inguinal lymph node (ILN) cells were detected 6 days after challenge infection. Immune cells ($1\times {10}^{6}$ cells in each tube) resuspended in PBS were incubated with stimulants at a concentration of 0.05 μg/100 μL of *P. berghei* antigen for 2 h at 37 °C or left unstimulated. The cells were incubated with the surface marker antibodies (CD3e-PE-Cy5, CD4-FITC, CD8a-PE, B220-FITC, GL7-PE; BD Biosciences, CA, USA) at 4 °C for 30 min. Stained cells were acquired using a BD Accuri C6 Flow Cytometer (BD Biosciences, CA, USA) and data were analyzed using C6 Analysis software (BD Biosciences, CA, USA).

### Determination of pro-inflammatory cytokines in spleen

At 6 days post-challenge infection, spleens of mice were collected and individually processed for splenocyte isolation as previously described [5]. Supernatants were collected after centrifugation at 2000 rpm for 10 min, and subsequently used to determine the level of inflammatory cytokines interferon-gamma (IFN-γ) and tumor necrosis factor-alpha (TNF-α) in spleen supernatant using the BD OptEIA IFN-γ and TNF-α ELISA kits (BD Biosciences, San Jose, CA, USA). Cytokine concentrations were assessed following the manufacturer’s instructions.

### Parasitemia

Infected mice blood samples were collected via retro-orbital plexus puncture every 3–5 days after challenge infection. RBCs were stained for parasitemia assessment as previously described [8]. Briefly, following infected blood collection, 2 μL of this blood was inoculated into a clean microcentrifuge containing 500 U/ml of heparin premixed in 100 μl of PBS. Afterward, blood samples were stained using 1 μl SYBR Green Ι (Invitrogen, Carlsbad, USA). Samples were incubated in the dark at 37 °C, 30 min, and flow cytometry was performed [8, 16].

### Statistics

All parameters were recorded for individuals within all groups. All data were presented as mean $\pm$ SD and statistical significances between groups were analyzed by one-way analysis of variance (ANOVA) and Student’s *t*-test using GraphPad Prism version 6.0 (San Diego, CA, USA). *P* values (*P < 0.05) were considered statistically significant.

## Results

### Construction and characterization of the P. berghei VLPs vaccine expressing codon-optimized AMA1 and MIC

**Fig. 1:**
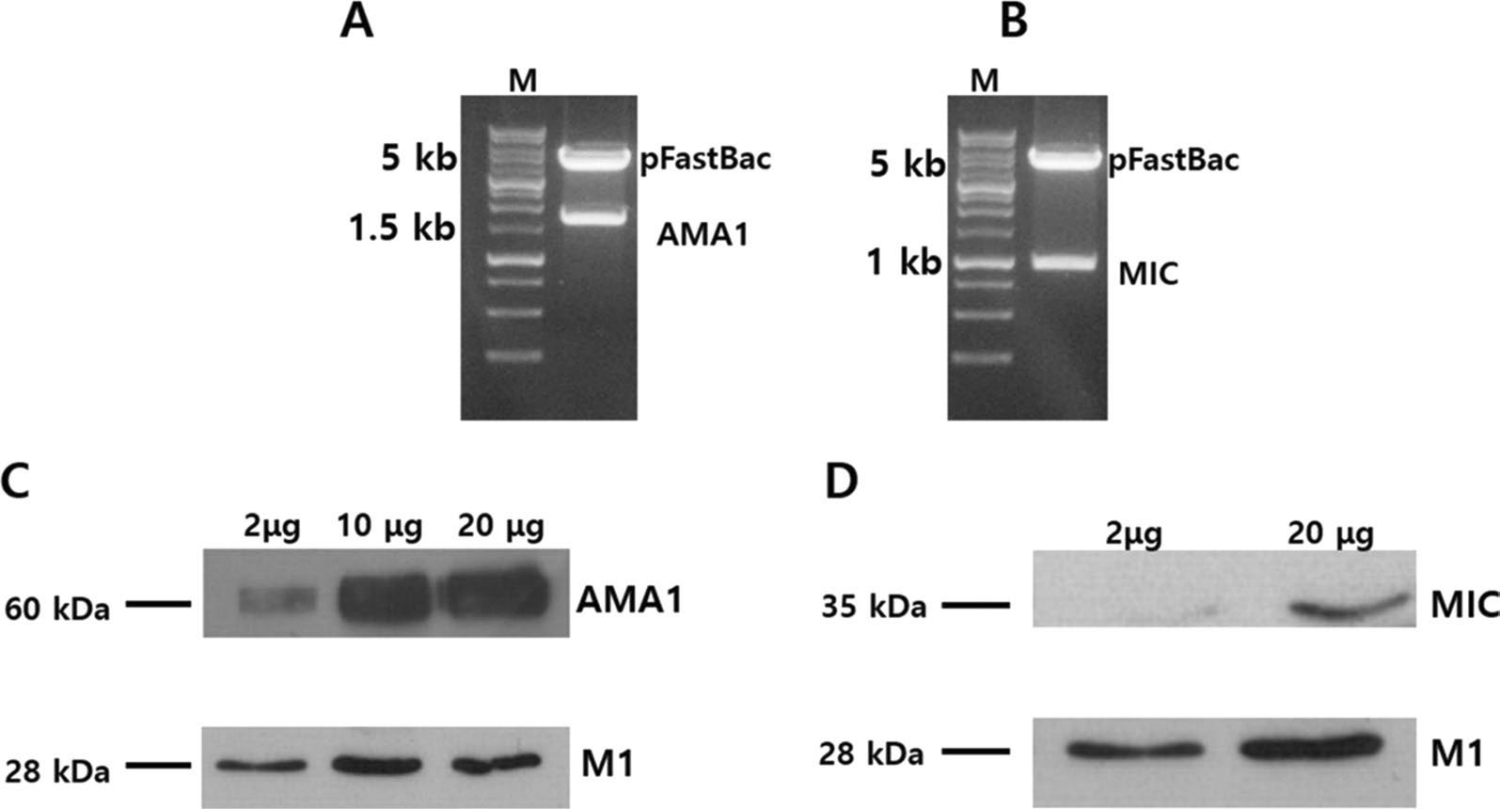
Characterization of *P. berghei* VLPs. To generate *P. berghei* VLPs containing AMA1 or MIC, rBVs for each antigen were generated using codon-optimized AMA1 or MIC genes. Genes were cloned into the pFastBac vectors and clones were confirmed by restriction enzyme cleavage with EcoRI/HindIII (**A**, **B**). M is DNA size marker (**A**, **B**). VLPs containing AMA1 or MIC were generated and characterized by western blot (**C**, **D**). AMA1 (60 kDa), MIC(35 kDa), and M1(28 kDa) proteins in the VLPs were determined as indicated (**C**, **D**)

Codon-optimized MIC was synthesized by GenScript, and it was confirmed that the translated amino acid sequence of the codon-optimized construct was identical to that of the original MIC amino acid sequence. Codon-optimized AMA1 was constructed as described previously [8]. The pFastBac-AMA1 and pFastBac-MIC plasmids were confirmed by restriction enzyme cleavage using EcoRI/HindIII (Fig. 1A, B). VLPs were generated by co-infecting Sf9 cells with rBVs expressing either *P. berghei* AMA1 or MIC with the influenza M1 following the procedure described previously [16]. Anti-*P. berghei* polyclonal antibody and anti-M1 monoclonal antibody were used to detect the expression of *P. berghei* AMA1, MIC, and influenza M1 in VLPs. The expressions of VLPs components were confirmed by western blot (Fig. 1C, D).

### IgG antibody responses were elicited following VLPs immunization

**Fig. 2:**
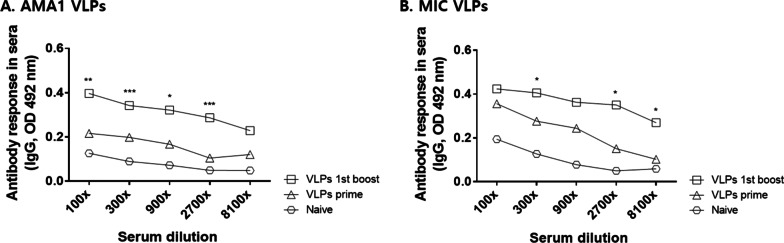
Confirming successful immunization of VLPs and antibody boosting effect. Sera of VLP-immunized mice were collected 4 weeks after prime and boost immunizations, and the IgG antibody reactions to VLPs were determined by ELISA. IgG antibody responses against AMA1 VLPs antigen (**A**) or MIC (**B**) were higher after boost compared to those after prime and naïve. Data are expressed as mean ± SD and statistical significance was denoted using an asterisk (*P < 0.05, ** *P* < 0.01, *** *P* < 0.001)

Sera were collected from mice 4 weeks after prime and the 1st boost immunization, and ELISA was performed to assess the level of IgG antibody response induced by VLP vaccinations. Compared to the naïve control mice sera, significantly higher levels of AMA1 and MIC-specific antibody responses were detected from immunized mice sera, with greater antibody levels being elicited following boost immunization than prime immunization (Fig. 2A, B). These results indicated that consecutive VLP immunizations successfully induced the boosting of antigen-specific antibody titers.

### *P. berghei*-specific IgG and IgG subclass responses in sera

**Fig. 3:**
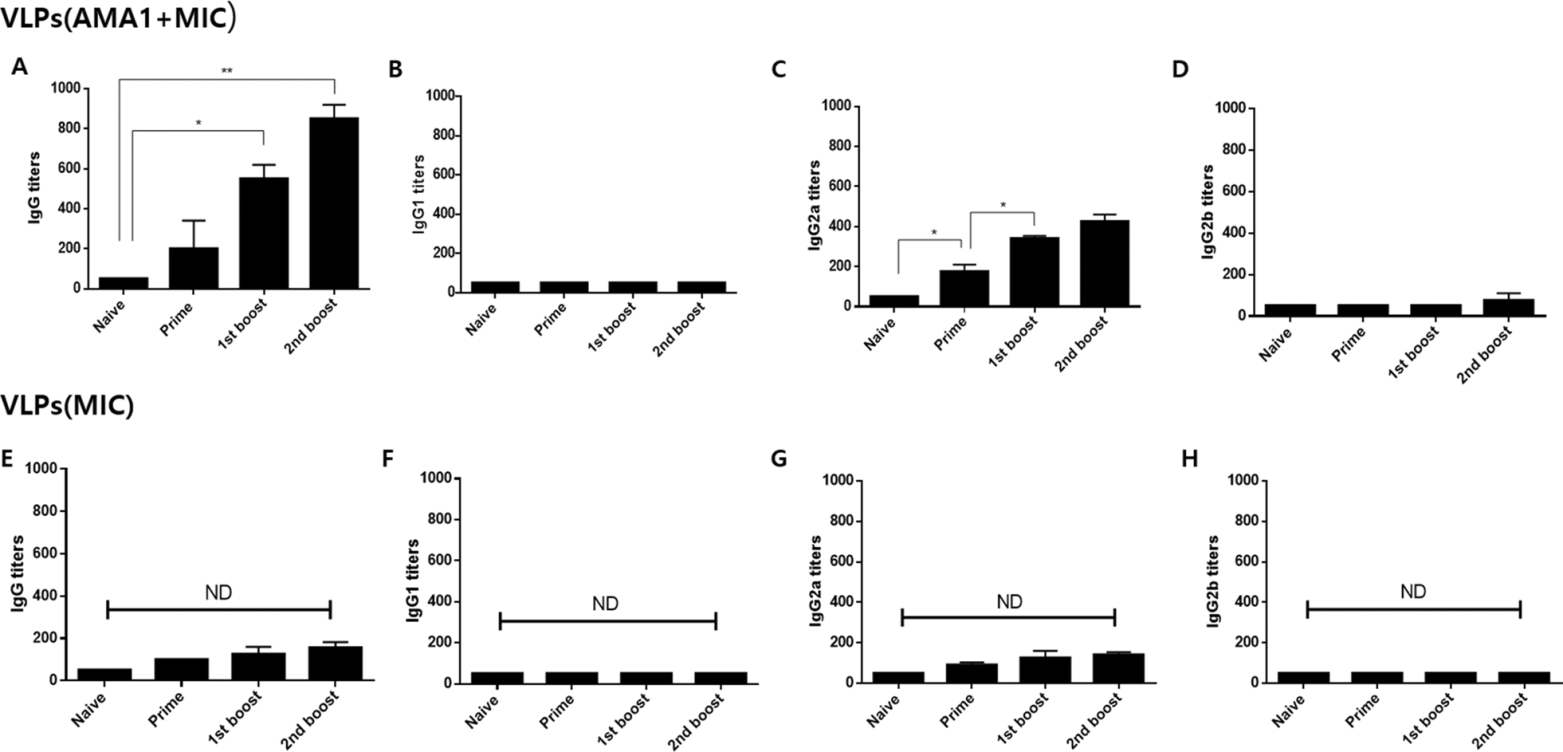
Parasite-specific IgG and IgG subclass antibody responses in sera. Parasite-specific IgG (**A**, **E**), IgG1 (**B**, **F**), IgG2a (**C**, **G**), and IgG2b (**D**, **H**) antibody responses were determined in VLPs (AMA1 + MIC) or VLPs(MIC)-immunized mouse sera 4 weeks after prime, 1st boost, and 2nd boost. Higher levels of *P. berghei*-specific IgG and IgG2a antibody responses were determined in the sera after prime, 1st boost, and 2nd boost compared to control. Data are expressed as mean ± SD and statistical significance was denoted using an asterisk (*P < 0.05). ND indicates no statistical significance among groups

*P. berghei-*specific IgG and IgG subclass antibody responses were determined from the sera of mice immunized with either VLPs (AMA1 + MIC) or VLPs (MIC). As seen in Fig. 3, for VLPs (AMA1 + MIC) immunization, parasite-specific IgG antibody responses were found to be higher after the 1st boost and 2nd boost immunizations compared to naïve (Fig. 3A, C, *p < 0.05). Significantly increased parasite-specific IgG2a antibody responses were observed after prime immunization, with each consecutive immunization inducing incremental enhancements to the IgG2a response (Fig. 3C, *p < 0.05). Compared to the naïve control, significant differences were not observed from IgG1 and IgG2b antibodies (Fig. 3B, D). On the contrary, compared to naïve control, VLPs (MIC) immunization-induced antibody responses were not significantly different across all immunization time points (Fig. 3E–H). These results indicated that only the VLPs containing both AMA1 and MIC elicited *P. berghei*-specific Th1-like antibody responses.

### T cell responses in the blood

**Fig. 4:**
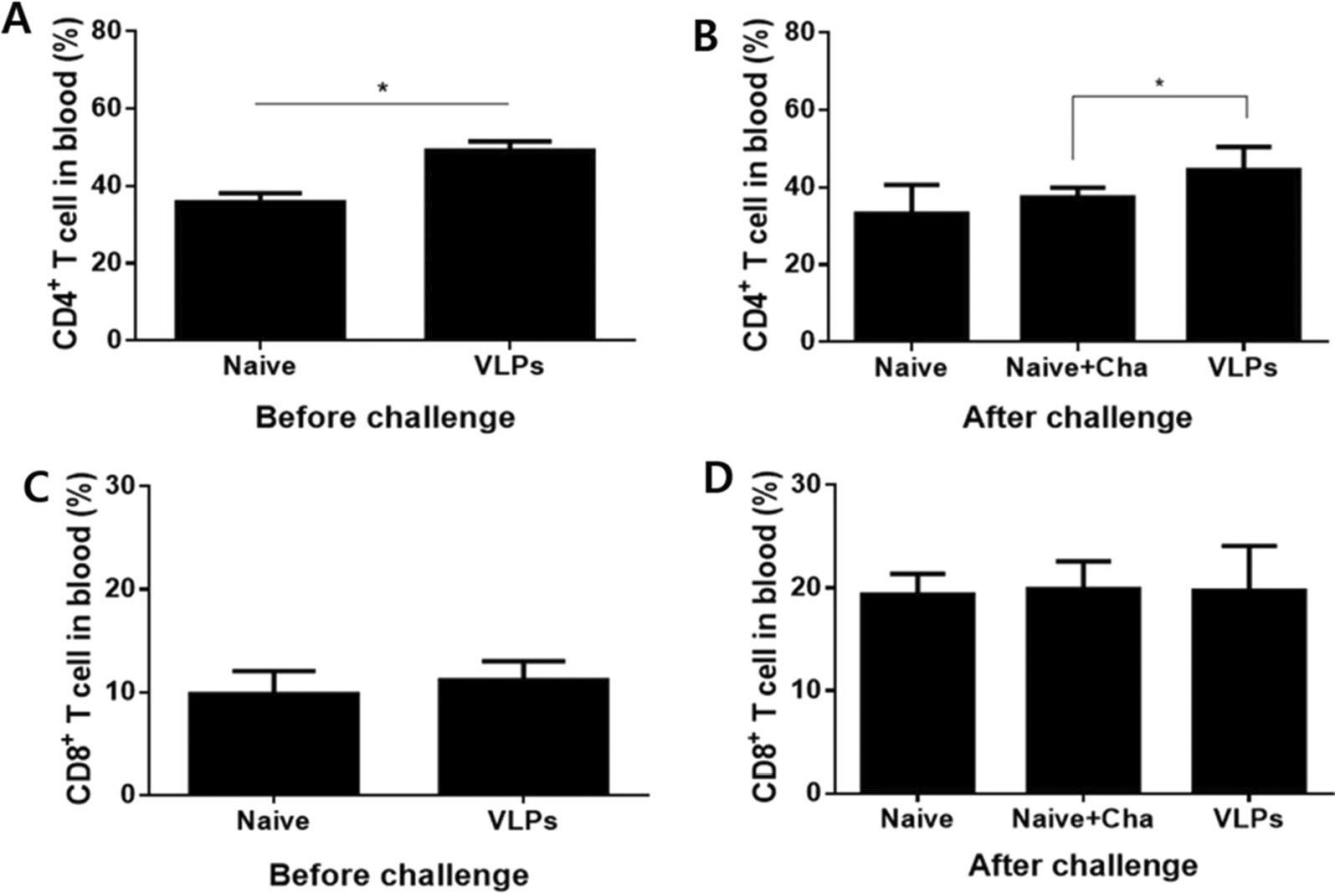
CD4^+^ T and CD8^+^ T cell responses in blood. Blood was collected to analyze CD4^+^ T and CD8^+^ T cell population 4 weeks after 2nd boost and on day 6 post-*P. berghei* infection by FACS. VLP immunization induced significantly higher levels of CD4^+^ T cell response before and after challenge infections compared to naïve or naïve challenge controls (**A**, **B**, *P < 0.05) whereas no significant increases in CD8^+^ T cell responses were observed among the three groups (**C**, **D**)

Blood CD4^+^ T and CD8^+^ T cell responses in mice immunized with VLPs before and after the challenge infections were determined by FACS analysis. As shown in Fig. 4, a higher level of CD4^+^ T cells was found in VLP-immunized mice before and after challenge infection compared to naïve control (Fig. 4A, B, *p < 0.05). However, changes to CD8^+^ T cell populations were not detected in the blood of mice before and after challenge infections (Fig. 4C, D). These results indicated that *P. berghei* VLP immunization induced CD4^+^ T cell response.

### CD4^+^ T, CD8^+^ T cells and germinal center B cell responses in the ILN

**Fig. 5:**
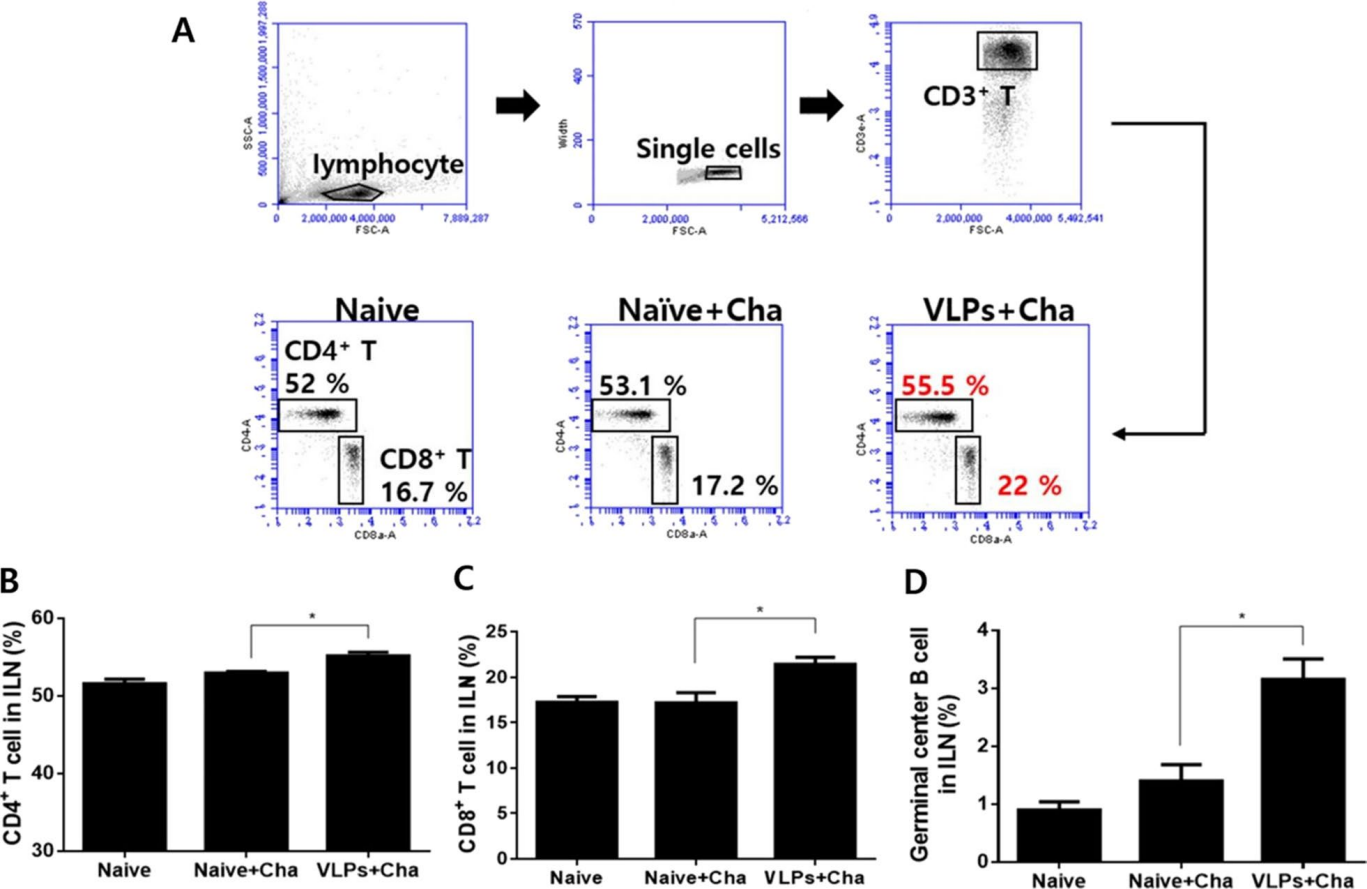
T and GC B cell responses in inguinal lymph node. At 6 days after *P. berghei* challenge infection, the inguinal lymph nodes (ILN) were collected to determine the levels of CD4^+^ and CD8^+^ T cell responses. The levels of CD4^+^ T and CD8^+^ T cells were determined through the gating strategy illustrated above (**A**). CD4^+^ T cells (**B**) and CD8^+^ T cells (**C**) in ILN cells were increased. Germinal center B cell responses in ILN cells were found to be higher compared to naïve and naïve challenge controls (**D**). Data are expressed as mean ± SD and statistical significance was denoted using an asterisk (*P < 0.05)

The levels of CD4^+^ T, CD8^+^ T cells, and germinal center B cell responses in the ILN of challenge-infected mice were determined by FACS analysis. Single cell population of lymphocytes was acquired and CD3^+^ cells were gated to assess the CD4^+^ and CD8^+^ T cell populations (Fig. 5A). Compared to the unimmunized controls, VLP immunization significantly enhanced the level of CD4^+^ T (Fig. 5B) and CD8^+^ T cells (Fig. 5C) in the ILN. Significantly higher levels of germinal center B cell responses in ILN cells were found in VLPs immunized mice compared to naïve challenge control (Fig. 5D). These results indicated that *P. berghei* VLP immunization induced germinal center B cell response and CD4^+^ and CD8^+^ T cell responses.

### CD4^+^ T, CD8^+^ T cells responses and pro-inflammatory cytokine responses in the spleen

**Fig. 6:**
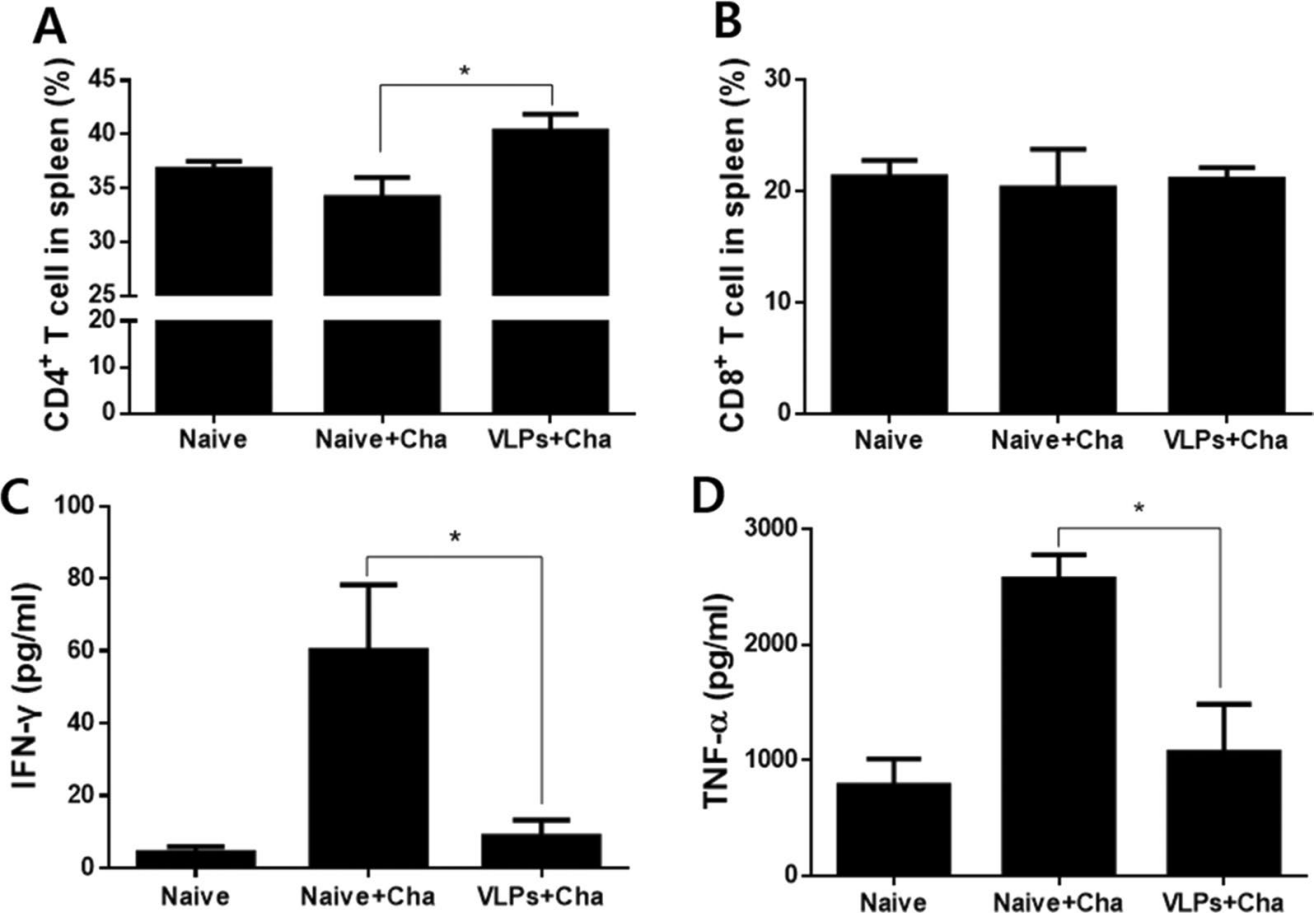
T cell response and pro-inflammatory cytokines in the spleen. Mice spleen were collected at 6 dpi, and the levels of CD4^+^, CD8^+^ T cells, and pro-inflammatory cytokines IFN-γ and TNF-α were determined. As shown in A and B, increased splenic CD4^+^ T cell populations were observed (**A**, *P < 0.05), whereas no changes were observed for CD8^+^ T cells (**B**). To determine the levels of pro-inflammatory cytokines IFN-γ and TNF-α, OptEIA IFN-γ and TNF-α ELISA kits (BD) were used. The levels of IFN-γ and TNF-α were significantly decreased in the VLP-immunized group compare to naïve challenge control (C, D, * *P* < 0.05)

To determine CD4^+^ T, CD8^+^ T cells responses and the pro-inflammatory cytokines IFN-γ and TNF-α in the spleen, spleen samples were collected from mice 6 days post-challenge infection. VLPs immunization induced higher levels of CD4^+^ T cell responses compared to naïve control (Fig. 6A, *p < 0.05), whereas no significant differences in CD8^+^ T cell responses were found (Fig. 6B). VLP immunization significantly reduced the levels of IFN-γ and TNF-α compared to naïve control (Fig. 6C, D, *p < 0.05). Consistent with the findings from ILN, these results indicated that VLPs containing both AMA1 and MIC induced CD4^+^ T cell responses, and lessened the production of inflammatory cytokines IFN-γ and TNF-α in the spleen upon *P. berghei* challenge infection.

### VLP vaccine efficacy in mice

**Fig. 7:**
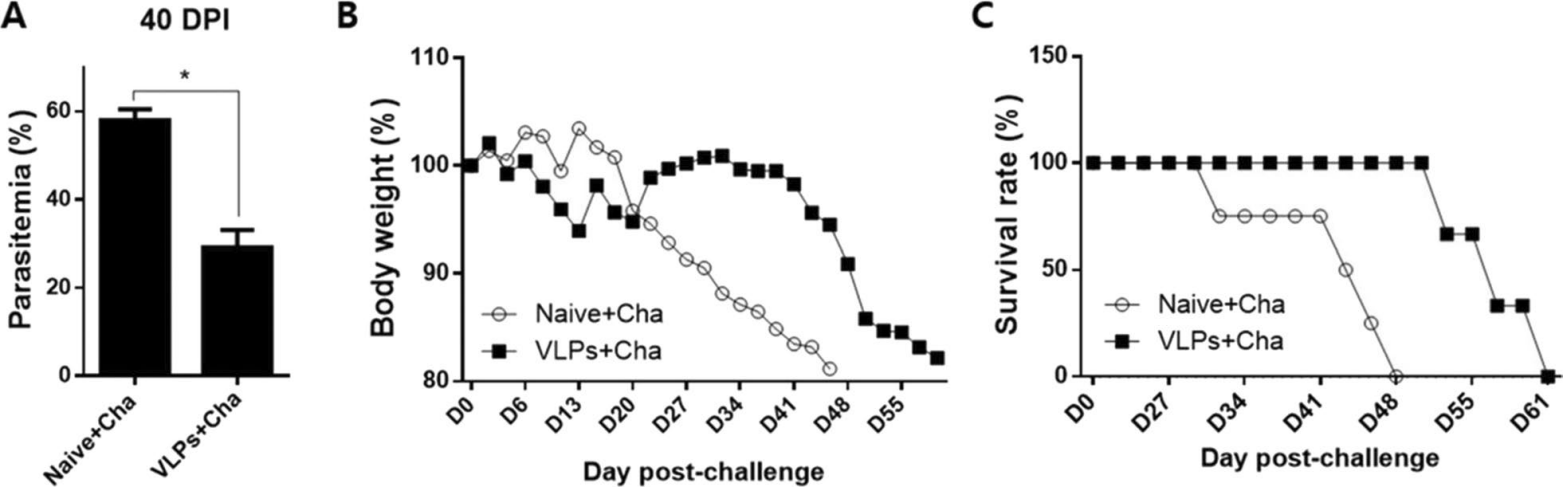
Protective efficacy of VLPs. At 4 weeks after the 2nd boost immunization, naïve and immunized mice were challenge-infected with 0.5% of *P. berghei* by intraperitoneal route. Mice were monitored for 61 days following *P. berghei* infection to determine parasitemia, bodyweight changes, and survival rate. A significantly lower level of parasitemia was detected at 40 dpi from VLPs (AMA1 + MIC)-immunized mice (**A**, *P < 0.05). Also, VLPs (AMA1 + MIC)-immunized mice were partially protected from bodyweight reduction (**B**) and survived longer (13 days) than naïve challenge control (**C**)

To determine the efficacy of the VLP vaccines, parasitemia in the blood, bodyweight reductions, and survival rates were determined post-challenge infection with *P. berghei*. Compared to unimmunized control, mice immunized with VLPs (AMA1+MIC) underwent nearly twofold parasitemia reductions at 40 days post-infection (dpi) (Fig. 7A). Unimmunized mice, gradual bodyweight loss became evident around 20 dpi, which continuously diminished until 48 dpi. Mice were protected from the bodyweight loss to a greater extent than unimmunized, but drastic weight loss became evident after 41 dpi (Fig. 7B). VLPs (AMA1+MIC)-immunized mice showed significantly enhanced survival compared to control (Fig. 7C). The VLPs (MIC)-immunized mice underwent continuous bodyweight reductions over time, a trend depicted by the unimmunized controls, and died on the same day as the unimmunized control (data not shown). AMA-1 VLPs immunization has shown the protection against *P. berghei* challenge infection [8]. These results indicated that VLPs (AMA1+MIC) immunization induced vaccine efficacy by lowing parasitemia, bodyweight loss, and longer survival time, whereas VLPs (MIC) immunization was not able to induce protection.

## Discussion

AMA1 is has been extensively studied as a potential blood-stage malaria vaccine candidate for its involvement in RBC invasion [17–19]. Our previous study indicated that the *P. berghei* VLPs vaccine expressing AMA1 induced humoral and cellular immunity in mice [8]. Interesting results involving microneme proteins have been reported from *Toxoplasma gondii*, a parasite belonging to the phylum Apicomplexa just as *Plasmodium* spp. VLP vaccine expressing the microneme protein 8 (MIC8) of *T. gondii* ensured the survival of immunized mice when challenge-infected with the virulent *T. gondii* RH strain [16]. This VLP vaccine, when co-administered with another VLP vaccine displaying *T. gondii* rhoptry protein 18 (ROP18) demonstrated significantly higher parasite inhibitory effect compared to either ROP18 or MIC8 VLP alone in the murine model [20]. In line with this notion, we hypothesized that influenza VLPs containing both AMA1 and MIC (VLPs (AMA1 + MIC)), could induce better vaccine efficacy against *P. berghei* challenge infection than single AMA1 or MIC containing influenza VLPs in mice. As expected, VLPs vaccine containing both AMA1 and MIC elicited parasite-specific IgG and IgG2a antibody responses, promoted germinal center B cell response, significantly reduced parasitemia, and prolonged the survival time of mice compared to mice immunized with AMA1 VLPs alone [8]. VLPs solely expressing the MIC antigen were unable to induce parasite-specific antibody responses or confer protection against *P. berghei* challenge infection, thus highlighting the importance of multi-antigenic VLPs for enhancing vaccine efficacy. Influenza M1 in VLPs has shown no virus-specific antibody response, neutralizing antibodies, and no lung virus reduction against live influenza virus challenge infection [21]. As such, M1 in VLPs may not contribute to the protection.

Contrary to the findings reported here, constructing vaccines based on the MIC proteins were proven to be highly successful in other apicomplexans such as *T. gondii* [22]. One potential explanation accounting for this difference can be attributed to the genetically divergent nature of these parasites. Evidently, despite belonging to the same phylum, only a limited number of proteins appear to be conserved between the two genera as multiple orthologs appear to be lacking [12]. For example, MIC8 protein appears to be a conserved feature shared across several apicomplexan parasites such as *T. gondii*, *Neospora*, and several others, but orthologs for this protein are absent in both *P. falciparum and P. berghei* [23]. Similarly, micronemal antigens such as the erythrocyte binding antigen 175 appear to be a specific feature of the *Plasmodium* spp. as its orthologs have not been identified in other apicomplexans such as *T. gondii*, *Cryptosporidium*, *Eimeria*, *Theileria*, and many others [23]. Based on this rationale, designing vaccines using the antigens that are conserved across the phylum could contribute to enhancing its protective efficacy.

The immunogenicity of codon-optimized AMA1 displayed using VLPs has been demonstrated in mice, as its immunization induced both humoral and cellular immunity [8]. Glycosylphosphatidylinositol (GPI)-anchored micronemal antigen (PvGAMA) from *P. vivax* has been shown to induce both humoral and cellular immune responses in individuals with naturally acquired *P. vivax* infection [9]

In the present study, both AMA1 and MIC were recognized by *P. berghei* antibody by western blot, which implies that both of these antigens were correctly expressed and reflect their native conformations [2, 3]. VLPs immunization elicited *P. berghei*-specific IgG and IgG2a antibody responses, indicating VLPs containing both AMA1 and MIC were involved in protection. Specifically, immunization with AMA1 and MIC VLPs combined significantly prolonged the survival time of immunized mice by 13 days, whereas the AMA1 VLPs reported in our previous study extended the survival period of immunized mice by 8 days [8].

One of the main factors contributing to protection against malaria infection is the presence of antibodies, which are essential for controlling the parasitic infection during the blood-stage of its life cycle [8, 24]. Previously, it has been reported that IgG2a is the most important IgG subclass antibody contributing to protection against *P. yoelii* infection in mice [25]. Using a transgenic *P. berghei* model, it was recently revealed that IgG1 subclass antibody may not be involved in malaria regulation [25, 26]. Consistent with these findings, in the current study, VLPs vaccine induced high levels of IgG2a isotype responses while no significant increases in IgG1 antibody responses were found, indicating IgG2a antibody mainly contributes to the parasitemia reduction. Importantly, mice immunized with the combination of malaria parasite antigen plus IL-12 in alum elicited higher levels of malaria parasite-specific IgG2a antibody response and protection compared to controls [27], supporting the findings of our study that VLP vaccination-induced IgG2a antibody response may provide protection against *P. berghei* infection.

CD4^+^/CD8^+^ T cells and germinal center B cells, along with antibody responses are important parameters for evaluating vaccine-induced protective efficacy [8]. In our present study, VLPs immunization significantly increased CD4^+^ T cells and GC B cell responses in blood, ILN, and spleen. Malaria infection incurs the exhaustion of both parasite-specific CD4^+^ and CD8^+^ T cells, which are mediated by the programmed cell death-1 (PD-1) signaling pathway [24]. This signaling pathway was responsible for the drastically reduced functional capacity of CD8^+^ T cells during the early stage of malaria infection and may account for the lack of sterile immunity observed post-clearance of acute infection [24]. In the current study, VLPs vaccine did not significantly increase the CD8^+^ T cells in the blood, ILN, and spleen, whereas CD4^+^ T cells in the blood, ILN, and spleen were found to be significantly increased, indicating that CD4^+^ T cells, not CD8^+^ T cells, may contribute to the protection against *P. berghei* infection.

In our previous study, VLPs immunizations from *Toxoplasma gondii* antigenic proteins or *P. berghei* MSP-8 and MSP-9 significantly lessened inflammatory cytokine responses [2, 5, 28]. Consistent with these findings, in our current study, VLP-immunized mice showed significant reductions in pro-inflammatory cytokines IFN-γ and TNF-α compared to the naïve challenge control, which may contribute to lessened parasitemia. The parasitemia, bodyweight changes, and survival are important parameters for vaccine efficacy evaluation. We found that VLPs expressing both AMA1 and MIC induced better protective efficacy than VLPs expressing AMA1 alone from our previous study by lowing parasitemia, lower bodyweight loss, and longer survival [8]. These results supported the previous finding that MIC is a novel blood-stage vaccine candidate antigen [10], and provided important information on malaria vaccine design strategy.

In summary, both AMA1 and MIC containing VLP vaccine, but not VLPs containing MIC alone, induced high levels of parasite-specific IgG and IgG2a antibody responses, CD4^+^, CD8^+^T cell responses, and GC B cell responses, significantly reducing parasitaemia, lessened bodyweight loss, and increased the survival rate. VLPs are effective vaccine candidates for controlling blood-stage infection of malaria.

## Supplementary Information

**Additional file 1. **Gene construct and VLP characterization.

## Data Availability

All data generated or analyzed during this study are included in this article.
