# Supplementary material for: Influenza virus-like particle vaccine containing both apical membrane antigen 1 and microneme-associated antigen proteins of Plasmodium berghei confers protection in mice
Source: BMC Immunol. 2022 Apr 25;23:21. doi: 10.1186/s12865-022-00494-4 (PMC9040335; doi:10.1186/s12865-022-00494-4)
Supplement: Supplementary file 1 — Additional file 1. Gene construct and VLP characterization. [file 12865_2022_494_MOESM1_ESM.docx]

**Supplementary Figure S1**

**
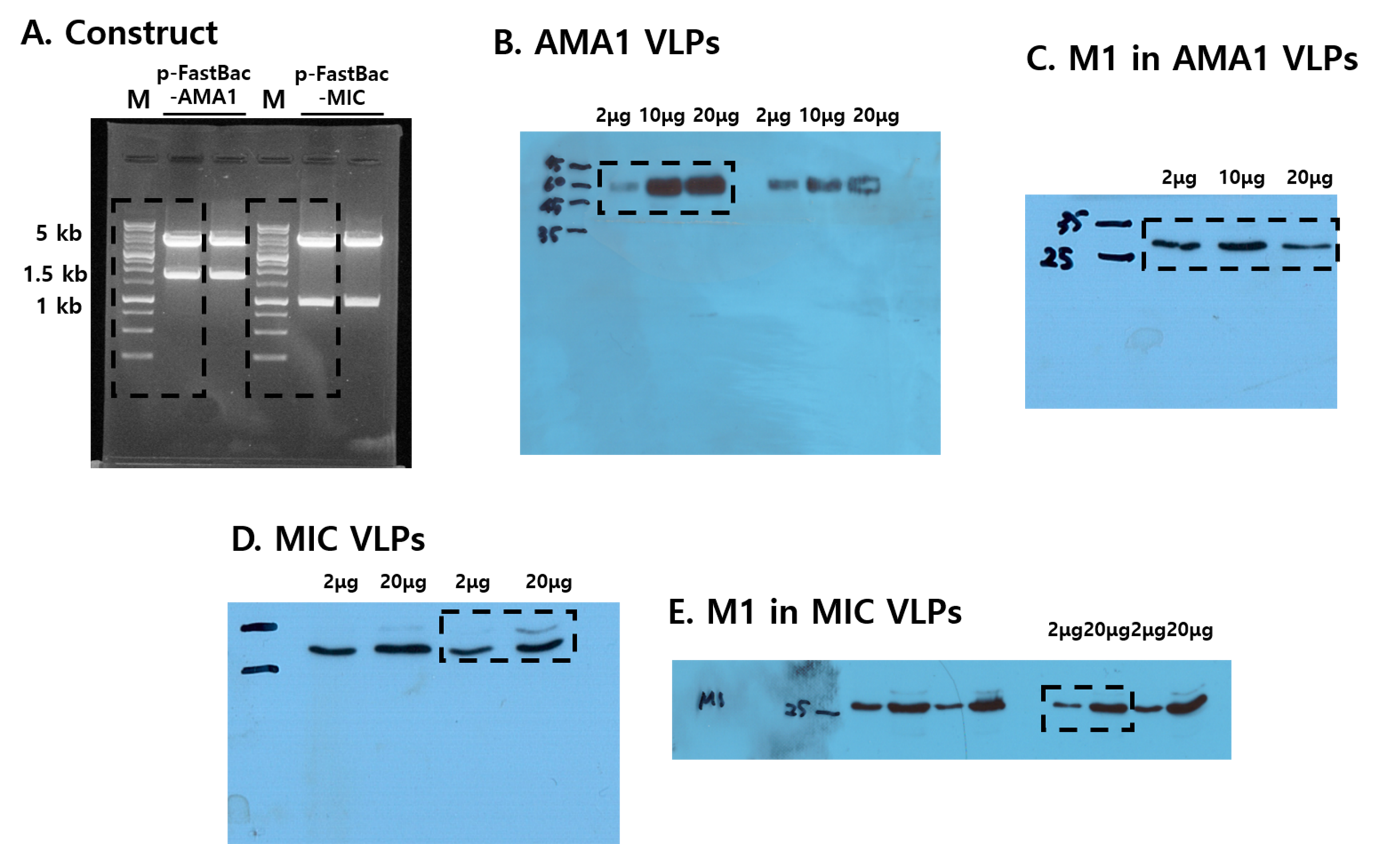
**

**Figure S1. Gene construct and VLP characterization**

AMA1 or MIC genes in pFastBac vector were digested by restriction enzyme cleavage with EcoRⅠ/HindⅢ (A). AMA1 in M1 VLPs (B, C) and MIC in M1 VLPs (D, E) were characterized by western blot.
